# Histomorphological patterns of regional lymph nodes in COVID-19 lungs

**DOI:** 10.1007/s00292-021-00945-6

**Published:** 2021-05-05

**Authors:** Jasmin D. Haslbauer, Matthias S. Matter, Anna K. Stalder, Alexandar Tzankov

**Affiliations:** 1 Pathology, Institute of Medical Genetics and Pathology, University Hospital Basel, University of Basel, Basel, Switzerland; 2 Institute of Medical Genetics and Pathology, Schönbeinstraße 40, 4031 Basel, Switzerland

**Keywords:** CD8-positive T‑lymphocytes, Cytokine release syndrome, Germinal center, Immunohistochemistry, SARS-CoV‑2, CD8-positive T‑Lymphozyten, Zytokin-Freisetzungssyndrom, Keimzentrum, Immunohistochemie, SARS-CoV‑2

## Abstract

**Background:**

A dysregulated immune response is considered one of the major factors leading to severe COVID-19. Previously described mechanisms include the development of a cytokine storm, missing immunoglobulin class switch, antibody-mediated enhancement, and aberrant antigen presentation.

**Objectives:**

To understand the heterogeneity of immune response in COVID-19, a thorough investigation of histomorphological patterns in regional lymph nodes was performed.

**Materials and methods:**

Lymph nodes from the cervical, mediastinal, and hilar regions were extracted from autopsies of patients with lethal COVID-19 (*n* = 20). Histomorphological characteristics, SARS-CoV‑2 qRT-PCR, and gene expression profiling on common genes involved in immunologic response were analyzed.

**Results:**

Lymph nodes displayed moderate to severe capillary stasis and edema, an increased presence of extrafollicular plasmablasts, mild to moderate plasmacytosis, a dominant population of CD8^+^ T‑cells, and CD11c/CD68^+^ histiocytosis with hemophagocytic activity. Out of 20 cases, 18 presented with hypoplastic or missing germinal centers with a decrease of follicular dendritic cells and follicular T‑helper cells. A positive viral load was detected by qRT-PCR in 14 of 20 cases, yet immunohistochemistry for SARS-CoV-2 *N*-antigen revealed positivity in sinus histiocytes of only one case. Gene expression analysis revealed an increased expression of *STAT1, CD163, granzyme B, CD8A, MZB1,* and *PAK1,* as well as *CXCL9*.

**Conclusions:**

Taken together, our findings imply a dysregulated immune response in lethal COVID-19. The absence/hypoplasia of germinal centers and increased presence of plasmablasts implies a transient B‑cell response, implying an impaired development of long-term immunity against SARS-CoV‑2 in such occasions.

## Abstract (de)

**Hintergrund:**

Eine dysregulierte Immunantwort, z. B. in der Form eines Zytokinsturmes, einer Störung des Immunglobulinklassenwechsels, eines sog. antikörpervermitteltem Enhancements oder einer aberranten Antigenpräsentation wurde bereits in schweren Krankheitsverläufen von COVID-19 beschrieben.

**Ziel der Arbeit:**

Zur Charakterisierung der COVID-19-Immunantwort wurde die Histomorphologie der Lymphknoten des pulmonalen Abflussgebietes untersucht.

**Material und Methoden:**

Regionale Lymphknoten des pulmonalen Abflussgebiets wurden bei COVID-19-Autopsien asserviert (*n* = 20). Deren Histomorphologie, SARS-CoV-2-qRT-PCR sowie Genexpressionsanalysen von gängigen Genen der Immunantwort wurden berücksichtigt.

**Ergebnisse:**

Histologisch zeigten sich ein mäßig- bis schwergradiges Ödem mit Kapillarostase, eine erhöhte Anzahl von extrafollikulären Plasmablasten, milde bis mäßige Plasmazytose, vermehrte CD8^+^-T-Zellen und CD11c/CD68^+^-Histiozyten mit Hämophagozytoseaktivität. Von 20 Fällen wiesen 18 hypoplastische oder fehlende Keimzentren sowie eine Verminderung der follikulären dendritischen Zellen und follikulären T‑Helferzellen auf. In 14 von 20 Fällen war der qRT-PCR-Nachweis von SARS-CoV‑2 positiv, jedoch zeigte sich nur bei einem einzigen Fall eine immunhistochemische Positivität für SARS-CoV-2-*N*-Antigene in Sinushistiozyten. In Genexpressionsanalysen war eine erhöhte Expression von *STAT1, CD163, Granzym B, CD8A, MZB1* und *PAK1*, neben *CXCL9* zu beobachten.

**Diskussion:**

Die Befunde in den Lymphknoten deuten auf eine dysregulierte Immunantwort bei schweren COVID-19-Krankheitsverläufen hin. Insbesondere impliziert das Ausbleiben der Keimzentrumsreaktion und die vermehrte Präsenz von Plasmablasten eine nur transiente B‑Zellreaktion, welche die Entwicklung einer Langzeitimmunität infrage stellt.

The year 2020 was markedly influenced by the coronavirus disease 2019 (COVID-19) pandemic caused by severe acute respiratory syndrome coronavirus 2 (SARS-CoV-2). Although the virus’ origin, cellular entry mechanisms, and epidemiology were rapidly elucidated [2, 18, 41], many fundamental immunopathological questions related to SARS-CoV‑2 remain unanswered.

In-situ analyses of viral pathophysiology thus remained at the level of small series and case reports [1, 30, 43], which were nonetheless instrumental in the understanding of this novel disease. For example, lymphopenia was one of the symptoms initially observed in cases with severe disease, its extent directly correlating with disease severity [16]. This was typically accompanied by an increased susceptibility to superimposed bacterial infections [30], but its morphological correlate in lymphoid tissues has not been fully elucidated.

Histomorphological patterns in lymph nodes [42] may provide valuable insights into the immunopathological mechanisms of disease. They reflect complex interactions between antigens, immune cells, signaling molecules (cytokines, chemokines, and growth factors), and the complement system [39]. Immunogenetics, such as gene polymorphisms of second messengers, receptors, and histocompatibility genes, often determines the severity and duration of the histomorphologically perceptible changes, as exemplified by sarcoidosis [14]. The most typical pattern of an adequate immune response is reflected by follicular hyperplasia with associated specific antibody production and establishment of an immunological memory [39]. Some other morphologic patterns also mirror signal transduction between messengers and cells. For instance, angiofollicular (Castleman/Castleman-like) hyperplasia is associated with chronic exposure to interleukin (IL)6 [12], while granulomas are associated with an overproduction of IL2, IL12, and tumor necrosis factor α (TNFα) [17]. Systemic lupus erythematosus, Kikuchi–Fujimoto disease, and toxoplasmosis show an increase in CD123/CD303^+^ plasmacytoid dendritic cells in lymph nodes due to the activation of Toll-like receptors 7 and 9 (TLR7, TLR9) and an overproduction of interferon γ (IFNγ) [20].

A somewhat less commonly considered histomorphological pattern in lymph nodes is the extrafollicular proliferation of B cells (B blasts) in the absence of follicular hyperplasia [5]. This is the morphological correlate of a rapid or primarily transient expansion of B cells without the formation of germinal centers. Histologically, lymph node architecture is intact; however, paracortical zones are replete with a polymorphic infiltrate of smaller blasts, centroblasts, immunoblasts, and—most instructively—plasmablasts. Immunophenotypic features of these blasts vary, showing heterogeneous positivity for CD20, CD30, CD38, CD79a, CD138, IRF4 (MUM1), and BLIMP1 [5]. They are polytypic for light chains and are 70–80% IgM^+^/CD27^−^/CD30^−^/CD79a^+^/CD138^−^, as they do not undergo an immunoglobulin class switch at the genetic level and do not experience a germinal center reaction at the physiological level. In 20–30% of cases, they are IgG^+^/CD27^+^/CD30^±^/CD79a^−^/CD138^+^, a likely immunophenotype of extrafollicularly activated marginal zone B cells without long-lasting B cell response [9]. A predominance of such extrafollicular B blasts would lead to an overarching production of low-affinity IgM antibodies without a lasting immunological memory. This pattern is predominantly observed in mucosal lymph nodes and splenic tissue.

This paper reviews current knowledge on immunopathological changes in COVID-19 and presents distinct histomorphological patterns of locoregional lymph nodes of COVID-19 lungs. These observations are discussed in the context of gene expression profiles and immunological phenomena in severe disease courses, as well as their potential role in the development of an effective immune response and immunological memory.

## Lymph node (histo)pathology of COVID-19

**Fig. 1:**
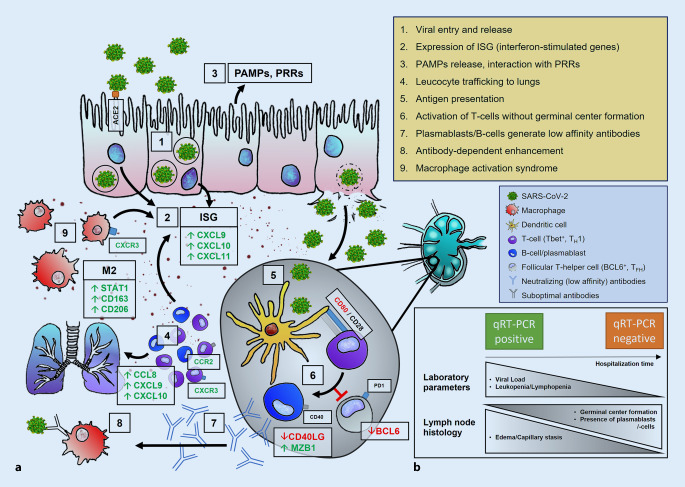
Immunopathology of SARS-CoV‑2 infection. **a** 1. Viral entry via ACE2, transcription of viral proteins and assembly, release of daughter virions. 2. Up-/dysregulation of ISG, cytokines, and chemokines. 3. Activation of PRRs by PAMPs. 4. Leukocyte trafficking into the lungs resulting in lymphopenia. 5. Antigen presentation in lymph nodes; activation of T cells (*purple*; e.g., by CTLA4/CD28) in a T_H_1 (Tbet^+^)-predominant milieu. 6. Inhibition of BCL6^+^ T_FH_ cells (*gray*) disrupts germinal center formation, resulting in the activation of extrafollicular B blasts and, thus, an increase in plasmablasts (*blue*). 7. Release of specific (low-affinity) antibodies (*blue*) against SARS-CoV‑2. 8. Antibody-dependent enhancement. 9. Macrophage activation syndrome with predominance of an M2 phenotype including HLH and cytokine storm. **b** Temporal evolution of laboratory parameters and histology in COVID-19 lymph nodes. *Short hospitalization time*: high viral load, severe lymphopenia, absence of germinal centers accompanied by severe edema/capillary stasis and plasmablast accumulation. *Longer hospitalization time*: lower viral load, regressing lymphopenia, incipient (delayed) germinal center formation. *ACE2* angiotensin-converting enzyme 2, *HLH* hemophagocytic lymphohistiocytosis, *ISG* interferon-stimulated gene, *PAMPs* pathogen-associated molecular patterns, *PRRs* pattern recognition receptors, *RT-PCR *reverse transcriptase polymerase chain reaction; *green* upregulated proteins, *red* downregulated proteins

An overview of the major pathophysiological mechanisms of the immune response in COVID-19 is presented in Fig. 1a.

### Lymphopenia

Severe lymphopenia was one of the first observations among COVID-19 patients in early clinical case series [16]; its severity was shown to be the most important predictive factor of disease progression in a meta-analysis of 32 studies involving over 10,000 patients [26]. Pathophysiologically, lymphopenia could be the outcome of chemotactic dysregulation of *CCL8, CCL20*, and *CXCL10*, which were shown to be upregulated in COVID-19 lung tissue [1]. These molecules are responsible for chemotaxis and migration of (T) lymphocytes, possibly leading to extensive leukocyte trafficking into the lungs. Furthermore, lymphocytes express angiotensin-converting enzyme 2 (ACE2) [46], rendering possible direct cytopathic effects a further plausible explanation of lymphopenia.

### Dysregulation of interferon-mediated signal transduction

IFN-mediated signal transduction plays a central role in the primary immune response to viral infections. Preliminary results of single-cell sequencing of peripheral blood mononuclear cells of COVID-19 patients revealed a phenotypic reconfiguration of immune cells with heterogeneous IFN-stimulated gene (ISG) signatures and downregulation of HLA class II genes, which was also associated with an increased plasmablast population [45]. Furthermore, ISG expression also appeared to be related to disease progression and viral load [34]. A subgroup of patients with early lethal disease showed high expression of ISG and cytokines, associated with high viral load and mild lung injury (ISG_high_) [30]. In contrast, another subgroup with prolonged disease showed decreased expression of ISGs (ISG_low_) in association with severe diffuse alveolar damage, lower viral load, and increased infiltration of CD8^+^ cells and (M2-polarized) macrophages [34].

### Dysregulation of antigen presentation

Gene expression analyses of COVID-19 lungs revealed downregulated expression of key costimulatory molecules of antigen presentation, such as *TLR7, TLR9*, and *CD86* [1]. CD86 is expressed by antigen-presenting cells and binds to CD28 or CTLA4 expressed by T cells. Interaction between CD86 and CD28 subsequently stimulates a T cell response [39]. Other TLRs (*TLR1, TLR4, TLR5*) were also found to be downregulated in COVID-19. While TLR1 binds to peptidoglycans of Gram-positive bacteria, TLR4 interacts with lipopolysaccharides (LPS) of Gram-negative bacteria and is central for recognition of the SARS-CoV‑2 spike antigen [36]. Activation of TLR5 by flagellin stimulates the innate immune response against certain bacterial species [39]. This may explain why COVID-19 patients experience increased susceptibility towards bacterial superimposition.

### Macrophage activation

Furthermore, the development of hemophagocytic lymphohistiocytosis (HLH) has been observed in COVID-19 patients [29, 38]. HLH typically is characterized by an uncontrolled macrophage activation syndrome, linked to impaired antigen presentation/recognition and excessive cytokine release (cytokine storm), but—to a limited and controlled extent—is known to be a typical reaction of the innate immune defense as a so-called pattern-recognition receptor (PRR)-associated response to pathogen-associated molecular patterns (PAMPs), specifically viral RNA [4, 30, 44]. HLH can be triggered by herpesviruses, especially EBV, but also by SARS-CoV‑1 and influenza viruses such as H5N1 and H1N1 [6, 15]. In accordance with this, COVID-19 tissue studies show increased expression of *CCL2 *and *CCL7* [1], known to be chemotactic for macrophages, thus stressing the central role of the cytokine storm in COVID-19 pathogenesis and highlighting a potential therapeutic intervention in severe disease [25]. Furthermore, an upregulation of genes encoding complement activation and phagocytosis is observed in severe disease [48]. In this context, pediatric COVID-19 patients have been observed to develop multisystemic hyperinflammation, which phenotypically resembles Kawasaki syndrome [37]. In such cases, severe vascular and cardiac manifestation was observed, occasionally together with macrophage activation syndrome.

### Dysregulation of the humoral immune response

In one of the first autopsy studies, an absence of germinal centers, an increased presence of plasmablasts, and intranodal capillary stasis were described in hilar lymph nodes and splenic parenchyma [30]. These histomorphological changes may be explained by a dysregulation of BCL6^+^ follicular T helper cells [21]which play an essential role  in germinal center functionality. Additionally, early blockade of T helper cell differentiation, a predominance of T‑bet^+^ T helper 1 cells, and an extrafollicular accumulation of TNFα could be shown [21], corresponding to a loss of follicular B cells by flow cytometric analyses of peripheral blood from severely ill COVID-19 patients [21]. Interestingly, TLR4 and TLR5, which are both downregulated in COVID-19 as mentioned earlier, are also essential for the germinal center response as they activate the NF-κB signaling pathway via MYD88 [13].

The previously mentioned proliferation of plasmablasts in hilar lymph nodes in lethal COVID-19 infection could represent a morphological correlate of a dysregulation of immunoglobulin (Ig) class switching. This is supported by a markedly increased plasmablast population in patients with severe disease progression, whereas a robust adaptive immune response with clonally expanded CD8^+^ effector- or emory cells, at best, is observed in mild disease [23, 49]. Gene expression analyses of COVID-19 autopsy tissue also demonstrated downregulation of *CD40LG* [1], which is an essential link of communication between T and B cells and significantly influences B cell maturation [39]. A defect in CD40LG results in an absence of Ig class switching, which may favor preferential extrafollicular proliferation of B cells. As a reflection of the aforementioned two features, in-depth immunological analyses demonstrated a negative correlation between the number of memory B cells and COVID-19 symptom duration [33]; the number of these cells correlated with IgG1 and IgM against the SARS-CoV‑2 spike protein. This was also reflected in antibody titer measurements [32] and flow cytometric analyses, which showed oligoclonal plasmablast expansion (>30% of circulating B cells) [22] in cases with severe disease. This, along with other immunologic signatures that correlated with disease progression, allowed a biostatistical classification of three COVID-19 immune phenotypes with different risk profiles [28].

As a link to immunopathology, an incomplete humoral immune response with low-affinity, non-(sufficiently)-neutralizing, low-titer antibodies can lead to antibody-dependent enhancement. This is defined as the generation of suboptimal antibodies, which enable virus penetration into Fc/complement receptor-bearing monocytes, macrophages, and granulocytes [19]. Indeed, data indicate an interaction of anti-spike protein antibodies and macrophages that contributes significantly to lung injury in SARS-CoV‑1 [24]. Finally, spike protein reactivity was also shown to be present in over one third of SARS-CoV-2-naïve patients. This implies the presence of cross-reactive T cells, developed in the course of immunization against other coronaviruses and may explain the more robust immune response in some patient groups [4].

## Methods

### Tissue extraction and histology

Hilar, mediastinal, and cervical lymph nodes were acquired from autopsies of COVID-19 patients (*n* = 20). Staining protocols of histochemical and immunohistochemical assays (H&E, IgG, IgM, CD3, CD11c, CD20, CD79a, CD68, CD163, CD206, HLA-DR, SARS-CoV-2‑N antigen [Rockland 200-401-A50 rabbit polyclonal antibody, dilution 1:2000]) were performed in accordance with accredited standard operating procedure (SOP) protocols of the Institute of Pathology in Basel. Histologic characteristics (number of plasmablasts, plasma cells, edema/capillary stasis, presence of HLH and germinal centers) were evaluated using ordinal scales (0–3; Table 1).

**Table 1:** Clinical, laboratory, and histopathological characteristics of qRT-PCR-positive and qRT-PCR-negative cases based on median viral load in lymph nodes

		qRT-PCR in lymph node		Total (*n* = 20)
		Positive (*n* = 14)	Negative (*n* = 6)	
*Clinical features/laboratory parameters (last value measured before death)*				
Hospitalization time (days; median; IQR)		6 (3–9)	11 (4–30)	8 (4–12)
CRP (mg/dl; median; IQR)		176 (100–271)	315 (232–337)	218 (144–280)
Leucocytes (10^9^/l; median; IQR)		8.1 (6.3–11.4)	16.0 (8.8–16.0)	8.8 (7.4–13.8)
Lymphocytes (10^9^/l; median; IQR)		0.7 (0.4–1.1)	0.5 (0.4–0.96)	0.7 (0.5–1.0)
Neutrophilic granulocytes (10^9^/l; median; IQR)		6.7 (3.7–10.1)	7.3 (7.26–10.6)	6.8 (6.3–10.1)
*Histopathology and postmortem qRT-PCR*				
Viral load, lungs (SARS-CoV‑2 genomes/10^6^ RNaseP copies; median; IQR)		2149 (57–60581)	123 (0–365)	146 (0–32218)
Viral load, lymph nodes (SARS-CoV‑2 genomes/10^6^ RNaseP copies; median; IQR)		117 (21–10392)	0 (0)	21 (0–3100)
Germinal centers (*n*, %)	Absent	9 (64)	3 (50)	12 (60)
	Scarce	4 (29)	2 (33)	6 (33)
	Extensive	1 (7)	1 (17)	2 (10)
Plasmablasts (*n*, %)	Absent/scarce	9 (64)	3 (50)	12 (60)
	Moderate	3 (21)	3 (50)	6 (33)
	Extensive	2 (14)	0 (0)	2 (10)
Plasma cells (*n*, %)	Absent/scarce	12 (86)	4 (66)	16 (80)
	Moderate	2 (14)	2 (33)	4 (20)
	Extensive	0 (0)	0 (0)	0 (0)
Edema and capillary stasis (*n*, %)	Mild	4 (29)	0 (0)	4 (20)
	Moderate	7 (50)	6 (100)	13 (65)
	Severe	3 (21)	0 (0)	3 (15)
Hemophagocytosis (*n*, %)	Absent	5 (36)	3 (50)	8 (40)
	Scarce	6 (43)	3 (50)	9 (45)
	Extensive	3 (21)	0 (0)	3 (15)

*qRT-PCR *quantitative reverse transcriptase polymerase chain reaction, *CRP* C‑reactive protein, *IQR* interquartile range

### RT-PCR

All lymph nodes underwent RT-PCR examination to determine viral load. The protocol was described in our previous study [30].

### Gene expression analyses

RNA was extracted by Forma HTG (HTG Molecular Diagnostics, Tucson, USA) from 10 µm thick untreated formalin-fixed paraffin-embedded (FFPE) blocks according to established protocols and processed and analyzed as described using the HTG EdgeSeq autoimmune assay [31].

### Statistics

Statistical analyses were performed using SPSS version 25 (IBM, Armonk, NY, USA). All correlation analyses were performed using Spearman rho (ρ).

## Results

Table 1 presents a summary of clinical and histological parameters of the 20 cases studied.

### Clinical parameters and RT-PCR

In 14 out of 20 cases, qRT-PCR detected SARS-CoV‑2 RNA. Interestingly, in cases without detectable SARS-CoV‑2 RNA, hospitalization time was longer (6 vs. 11 days), suggesting a biphasic disease course with progressive viral elimination, similar to previously performed analyses of the same autopsy cohort ([34]; Fig. 1b). This is supported by a negative correlation of pulmonary viral load with hospitalization time (ρ = −0.776; *p* < 0.0001) and a positive correlation between pulmonary and lymph node viral load (ρ = 0.514; *p* = 0.035). Blood counts revealed a positive correlation between hospitalization time and absolute lymphocyte count (ρ = 0.582; *p* = 0.014) and a negative correlation with the absolute leucocyte count (ρ = −0.577; *p* = 0.015).

### Histopathology

**Fig. 2:**
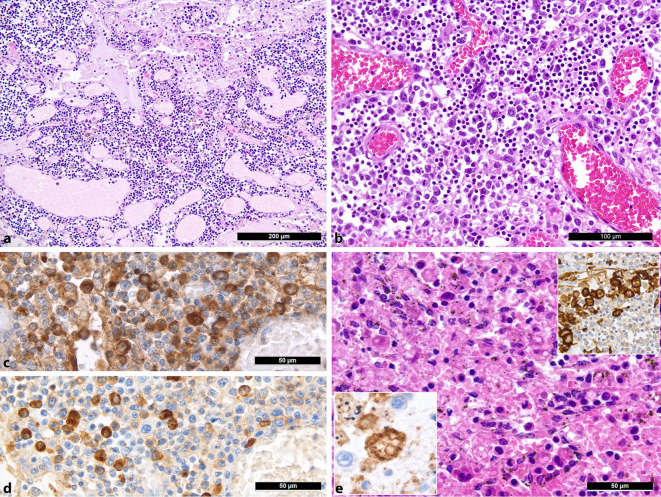
Histomorphological characteristics of regional lymph nodes of COVID-19 lungs. **a** Moderate to severe edema with capillary stasis (H&E; 50 ×). **b** Proliferation of extrafollicular plasmablasts (H&E; 200 ×). **c, d** IgG (**c**) and IgM (**d**) staining of plasmablasts (immunoperoxidase; 400 ×). **e** Hemophagocytosis in the sinus of a lymph node (H&E; 360 ×). *Left inlet*: positivity for CD11c in histiocytes undergoing hemophagocytosis (immunoperoxidase; 400 ×); *right inlet*: positivity for CD206, a specific marker of M2 polarization [48], in sinus histiocytes

Major histologic lymph node features included moderate-to-severe edema and capillary stasis (Fig. 2a) in all cases, explained by acute right heart failure due to severe pulmonary disease. Further findings include a proliferation of extrafollicular B blasts, IgG- and IgM-positive plasmablasts in particular (Fig. 2b,c; 12/20), consistent with rapid or primarily transient B cell immune response bypassing the germinal center response as described above [5], with largely absent or hypoplastic/hypotrophic germinal centers or secondary follicles (12/20), including decreased follicular dendritic cells and follicular T helper cells. Correlation analyses showed a negative association between the presence of secondary follicles and viral load in the lungs (ρ = −0.645; *p* = 0.005) and CRP (secondary follicles = −0.522; *p* = 0.032), which, in conjunction with previous analyses of the same autopsy cohort [34], additionally underscores a delayed germinal center response. Furthermore, a discrete-to-moderate degree of plasmacytosis and a predominance of CD8^+^ T cells was observed, accompanied by numerous HLA-DR-, CD163-, and CD206-positive M2-polarized macrophages [24], CD11c- and CD68-positive histiocytes, and the presence of hemophagocytosis in the sinuses in 12/20 cases (Fig. 2d), consistent with the aforementioned phenomena of macrophage activation in COVID-19 [29, 38]; however, the full clinical picture of HLH presented itself in only one of the 20 patients examined here [47].

**Fig. 3:**
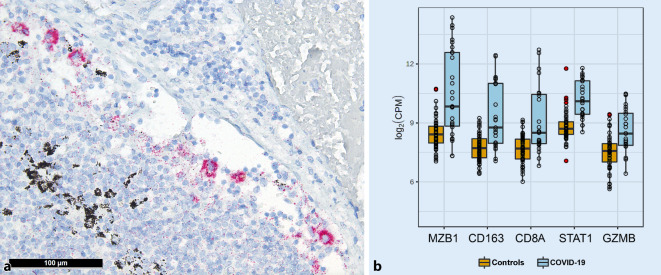
Immunohistochemistry detecting SARS-CoV‑2 and associated gene expression profiles in COVID-19. **a** Positivity in sinus histiocytes for SARS-CoV‑2 as detected by immunohistochemistry for the SARS-CoV-2 N-antigen (immunoperoxidase with 3‑amino-9-ethylcarbazole used as a chromogen; 200 ×). **b** Gene expression profile shows significantly increased expression of *MZB1, CD163, CD8A, STAT1*, and *GZMB* in COVID-19 compared to controls

An increased number of sinus histiocytes with corresponding positive immunohistochemical detection of the nucleocapsid antigen of SARS-CoV‑2 in an elderly patient with a 30-day disease course may be interpreted as antibody-dependent enhancement, as further supported by observations from Martines et al. ([27]; Fig. 3a). Although the majority of lymph nodes examined illustrated imperceptible virus antigen levels as detected by immunohistochemistry, the lungs of the same patient showed a comparatively higher viral copy number than others (990 viral genome copies/10^6^ human RNAseP copies in the lymph nodes and approximately 125000 in the lungs).

### Gene expression profiles

In line with the histomorphological patterns described above, gene expression profiles (Fig. 3b) showed increased expression of the following genes: *STAT1* (central transcription factor in macrophage activation), *CD163* (hemoglobin–haptoglobin complex receptor and marker of M2 macrophage polarization [24]), granzyme B (*GZMB*) but not perforin (consistent with the imbalance of both proteins in hemophagocytic lymphohistiocytosis [40]), *CXCL9* and *PAK1* (a chemokine and an enzyme important for the migration of cytotoxic T cells), *CD8A *(in line with the upregulation of* PAK1*), and, finally, *MZB1 *(marginal zone B and B1 cell-specific protein, which contributes to the composition and secretion of IgM and thus corresponds with the increased plasmablasts).

## Discussion

The histomorphological patterns observed in lymph nodes appear to reflect the underlying immunopathology of lethal COVID-19. Our data imply a biphasic disease course, demonstrated by an initially high viral load and severe lymphopenia, which both eventually regress. Histologically, edema and plasmablast activation are evident in the lymph nodes of early lethal cases, followed by macrophage activation and a subtle germinal center reaction in cases with a longer disease duration (Fig. 1b), thus supporting this premise. These data are consistent with gene expression analyses performed on the same autopsy cohort [34], which unraveled a marked variance in ISG gene signature in patients with varying hospitalization time. A disruption of the IFN response is implied in severe COVID-19, as supported by the following:

1. All coronaviruses, especially SARS-CoV‑2, suppress the production and release of all three types of interferons [10].
2. Patients with severe COVID-19 show loss-of-function variants in TLR- and interferon-dependent genes or neutralizing antibodies to type I IFN (α and ω) [3, 49].
3. Dysregulation of type I IFN seems to be generally crucial for disease progression [7, 8].
4. The ISG response changes significantly as COVID-19 progresses and seems to influence different aspects of immunopathology [34].
  This disruption of the IFN response may explain the dysregulation of BCL6^+^ follicular T helper cells [21], the rapid but poorly specific B cell immune response bypassing the germinal center reaction with plasmablasts generating low-affinity antibodies [5, 22, 45], the resultant antibody-dependent enhancement with macrophage (hyper)activation [19] with sinus histiocytosis and possibly HLH [29, 38], and M2 macrophage polarization [24, 34, 49]. This ultimately results in a highly pathogenic inflammatory monocyte-macrophage response that is well documented in SARS-CoV‑1 and MERS [7, 8, 24], leading to extensive organ damage [25, 48]. Antibody-dependent enhancement could also contribute to the in-situ positivity in monocytes/macrophages (Fig. 3a; [19]) and thus, by extension, systemic viral spread.

These complex immunopathological observations, illustrated in part by the histopathologic changes described in this study, could also explain the underlying challenges in efficient coronavirus vaccine development [11]. Furthermore, findings from in-situ studies of the lymphoid compartment in COVID-19 could provide valuable pharmacological approaches, such as TLR agonists [35], to increase the efficiency of vaccines under development, thus ensuring a durable immune response and memory.

## Practical conclusion

- The analysis of histopathological features in locoregional lymph nodes is indispensable for the understanding of COVID-19 pathophysiology. In particular, the absence of a germinal center response accompanied by plasmablast expansion and a lack of immunoglobulin class switching suggest an inefficient antibody response in patients with severe disease.
- A dysregulation of interferon (IFN)-mediated signal transduction appears to be central to the immunopathology of COVID-19. Initial therapeutic approaches of IFN I‑interfering drugs (JAK inhibitors such as baricitinib or dexamethasone) are currently undergoing clinical trials (NCT04358614), while potential clinical studies involving TLR agonists are yet to be launched.
- Further studies are needed to shed light on the underlying mechanisms of innate and humoral immune responses in mild and severe disease courses, as well as phenomena such as cross-reactivity of certain T cell subpopulations. This will potentially aid the development of an efficient vaccine.

## Abbreviations

BLIMP-1/PRDM‑1: PR domain zinc finger protein 1
CCL: Chemokine ligand
CD: Cluster of differentiation
COVID-19: Coronavirus disease 2019
CTLA4: Cytotoxic T lymphocyte-associated protein 4
CXCL9: Chemokine (C-X‑C motif) ligand 9
EBV: Epstein–Barr virus
Fc: Fragment constant
HLA-DR: Human leukocyte antigen DR
HLH: Hemophagocytic lymphohistiocytosis
IFN: Interferon
Ig: Immunoglobulin
IL: Interleukin
IRF4 (MUM-1): Interferon regulatory factor 4
ISG: interferon-stimulated genes
LPS: Lipopolysaccharide
MYD88: Myeloid differentiation primary response 88
MUM‑1 (IRF4): Multiple myeloma 1 protein
MZB1: Marginal zone B and B1 cell-specific protein
NF-κB: Nuclear factor kappa B
PAK1: P21 (RAC1)-activated kinase 1
PAMPs: Pathogen-associated molecular patterns
PRRs: Pattern recognition receptors
qRT-PCR: Quantitative reverse transcriptase polymerase chain reaction
SARS-CoV-1/2: Severe acute respiratory syndrome coronavirus 1/2
STAT1: Signal transducer and activator of transcription 1
TLR: Toll-like receptor
TNF‑α: Tumor necrosis factor alpha

## Declarations

All procedures performed in studies involving human participants or on human tissue were in accordance with the ethical standards of the institutional and/or national research committee and with the 1975 Helsinki declaration and its later amendments or comparable ethical standards. Informed consent was obtained from all individual participants included in the study.

The supplement containing this article is not sponsored by industry.
